# Conserved glycan-utilization strategies shape *Akkermansiaceae* success across aquatic and gut ecosystems

**DOI:** 10.1093/ismejo/wrag096

**Published:** 2026-04-22

**Authors:** Isabella Wilkie, Nicole Von Possel, Tomás Sauma-Sánchez, Greta Reintjes, Luis H Orellana

**Affiliations:** Ecological Genomics Group, Max Planck Institute for Marine Microbiology, 28359 Bremen, Germany; Ecological Genomics Group, Max Planck Institute for Marine Microbiology, 28359 Bremen, Germany; Ecological Genomics Group, Max Planck Institute for Marine Microbiology, 28359 Bremen, Germany; Microbial-Carbohydrate Interactions Group, Faculty of Biology/Chemistry, University of Bremen, 28359 Bremen, Germany; Ecological Genomics Group, Max Planck Institute for Marine Microbiology, 28359 Bremen, Germany

**Keywords:** Akkermansiaceae, microbial ecology, Verrucomicrobiota, microbiome, microbial evolution, host-associated

## Abstract

Elucidating interaction mechanisms and substrate specialization is central to understanding bacterial adaptation across ecological niches. Specialized mucin-degrading bacteria of the genus *Akkermansia* are widely recognized for their beneficial roles in the human gut, yet it remains unclear whether this specialization is unique to the gut or reflects a conserved ecological strategy across different hosts and environments. Here, we show that members of the family *Akkermansiaceae* share a deeply conserved genetic and mechanistic framework, enabling colonization across gut and aquatic ecosystems. Comparative genomics of *Akkermansiaceae* representatives revealed niche-specific gene repertoires tightly adapted to substrate source and availability. Marine representatives encode distinct combinations of CAZymes and comparatively expanded sulfatase repertoires that enable the degradation of sulfated polysaccharides such as fucoidan, a recalcitrant substrate linked to carbon sequestration. Structural predictions and comparisons identified a conserved molecular system centered on a type IV–like pilus that mediates attachment to complex, fucose-rich glycans. The genes underlying this system are syntenic with the recently described mucin utilization locus in *Akkermansia muciniphila*, revealing an evolutionary continuity between aquatic and gut lineages. Seawater incubations with fluorescently labeled substrates confirmed fucoidan uptake and degradation by marine *Akkermansiaceae*. Together, these results reveal a unified glycan-utilization strategy spanning the environmental breadth of *Akkermansiaceae* and provide a mechanistic framework linking ecological success in marine environments to traits associated with probiotic functions in the human gut.

## Main

**Figure 1:**
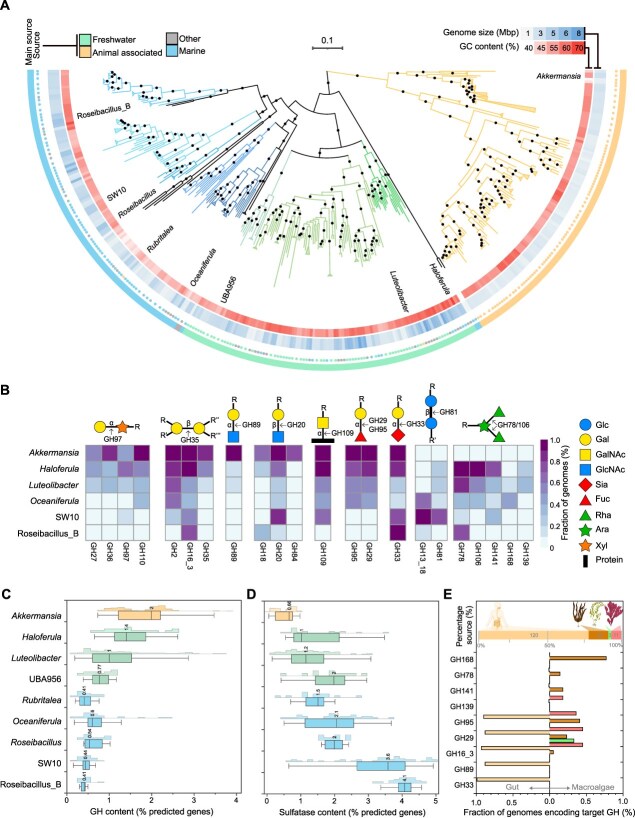
Members of family *Akkermansiaceae* specialize in degrading fucose-containing substrates across environments. **A.** Phylogenetic reconstruction from genomes and MAGs (genome size ≤8 Mbp; see extended results). Bootstrap values >95% (black dots). Genus-level taxonomy is indicated by branch color and name. Taxonomic assignments follow GTDB R220 (see extended results for recent reclassifications). **B.** GH families potentially involved in degrading fucose-containing substrates. **C-D.** Fraction of GHs (**C.**) and sulfatases (**D.**) across marine, freshwater, and gut genomes. **E.** Selected GHs in host-associated *Akkermansiaceae.*

The phylum *Verrucomicrobiota* is typically underrepresented, partly due to relatively low abundances and isolation difficulty. Most research focuses on the family *Akkermansiaceae*, particularly *Akkermansia muciniphila* [1–5], a mucin-colonizing gut bacterium associated with human health [6, 7]. These species specialize in degrading mucins (glycosylated proteins) in the mucus layer covering the intestinal epithelium. Mucin degradation requires the removal of protective fucose and sialic acid caps [8], and the subsequent breakdown of highly *O*-glycosylated proteins (e.g. Fig. 1B). Metagenomic profiling via Sandpiper [9] (see methods), comprising ~248 000 publicly available metagenomes, confirmed the predominance of *Akkermansiaceae* in animal guts (identified in 26 033 metagenomic datasets) and revealed their presence in marine (5560) and freshwater (1245) environments. Within the family, the genera *Haloferula* and *Luteolibacter* were predominant in freshwater, whereas *Oceaniferula* and *Roseibacillus* dominated marine ecosystems. We analyzed 346 high-quality and 98 medium-quality *Akkermansiaceae* genomes and metagenome-assembled genomes (MAGs). Phylogenetic clustering corresponded closely to genome source at the genus level (Fig. 1A). Although these bacteria are widespread across diverse environments, the lifestyle of aquatic *Akkermansiaceae* and the mechanisms driving their evolutionary adaptations remain poorly understood. We hypothesize that cellular strategies for substrate utilization and host interaction mechanisms known in gut *Akkermansiaceae* share a blueprint with their aquatic relatives. We integrated cell-level phenotyping with analyses of gene organization and protein structure prediction from environmental *Akkermansiaceae* to examine genetic adaptations driving substrate specialization across environments.

Representative *Akkermansiaceae* genomes and MAGs derived from gut and aquatic environments harbored unique gene combinations indicative of adaptations to substrates prevalent in their habitats (Fig. 1B; Table S1). Most genomes (396/444) encoded enzymes targeting fucose-containing glycans (such as those of glycoside hydrolase (GH) family 29, GH78, GH95, GH106, GH139, GH141, GH151, GH168, or GH174). However, their distribution followed habitat-specific patterns. The co-occurrence of α/β-galactosidases, α/β-N-acetylglucosaminidases, sialidases, and fucosidases, a signature of mucin degradation, was characteristic of gut-associated *Akkermansiaceae*, highlighting the adaptation to host-derived glycans (Fig. 1B and E). By contrast, their reduced presence in aquatic groups indicates divergent glycan utilization strategies. For instance, only macroalgae-associated *Akkermansiaceae* (36/156 genomes) contained GHs associated with the degradation of algae-derived fucose-containing sulfated polysaccharides (FCSPs) (GH78, GH139, GH168, and GH141) (Fig. 1B and E). The consumption of methyl pentoses in *Akkermansiaceae* is compartmentalized in bacterial microcompartments (BMCs) [10, 11], which were only detected in aquatic representatives (Table S1, *n* = 99). BMCs are central for detoxifying lactaldehyde, the intermediate of fucose and rhamnose degradation [11]. Marine genomes had the highest fraction of sulfatases, ranging from 1.5% to 4.1% of total genes depending on the genus (Fig. 1D). In contrast, gut-derived genomes contained the lowest fraction of sulfatases, but a higher fraction of GHs than freshwater and most marine genomes, consistent with the expectation that gut substrates are less sulfated, yet enriched in complex glycan linkages. In aquatic *Verrucomicrobiota*, BMCs coupled with fucose-degrading enzymes likely enable the utilization of recalcitrant substrates while avoiding strong competition for simpler polysaccharides, as observed during spring blooms in the North Sea [12]. Size-fractionated metagenomes [13] (a proxy for lifestyle differentiation) revealed that *Akkermansiaceae* members were on average ~ 8x more abundant in particles (≥3 μm) than in free-living fractions (Fig. S1, Fig. S2, Table S3), indicating physical niche differentiation and a surface-associated lifestyle.

**Figure 2:**
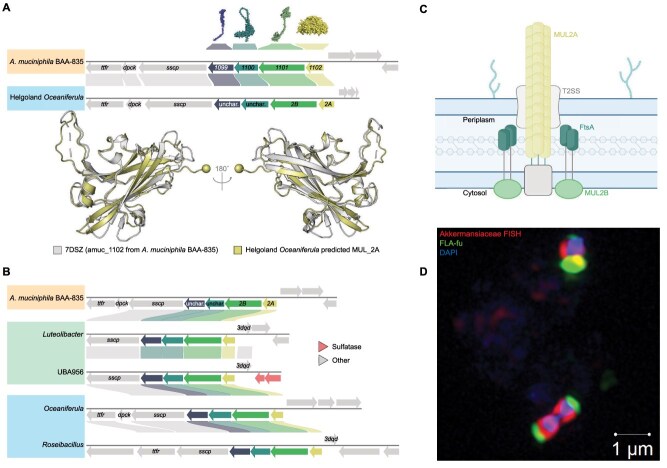
Aquatic *Akkermansiaceae* lifestyle and proposed substrate uptake mechanisms. **A.** Predicted structure of MUL2A monomer from an *Oceaniferula* MAG (Helgoland) superimposed on the experimental amuc_1102 structure (7DSZ). The *MUL2* genetic region and synteny are shown for *Oceaniferula* and *A. muciniphila* genomes and the predicted structures of *A. muciniphila* amuc_1099–1101 and amuc_1102 homotrimer. **B.** Synteny of *MUL2* loci in representative gut, freshwater, and marine genomes. Others: Secretion system channel protein (sscp), dephospho-CoA kinase (dpck), transcription termination factor rho (ttfr), and 3-dehydroquinate dehydratase (3dqd). **C.** Predicted MUL2 complex involved in pilus biogenesis for attachment to particles (adapted from Davey et al. (2023) and Hughes et al. (2025)). **D.** Photomicrograph obtained using super resolution structured illumination microscopy (SR-SIM) showing DAPI-stained cells, FISH-labelled *Akkermansiaceae*, and fluorescently labelled fucoidan (FLA-fu).

The mechanisms underlying substrate acquisition in aquatic *Akkermansiaceae* are largely understudied. In *A. muciniphila*, two mucin utilization loci (*MUL1* and *MUL2*) mediating the transport and processing of mucin were recently identified [4]. *MUL2* likely facilitates attachment to surfaces similar to a secretion system (Fig. 2D), whereas *MUL1* resembles the Sus system in *Bacteroidota* [4, 14]. The absence of *MUL1* synteny in aquatic *Akkermansiaceae* genomes suggests that it represents a mucin-specific adaptation in gut-associated lineages [4, 14]. The *MUL2* locus comprises four genes: *amuc_1099*, *amuc_1100* (both uncharacterized), *amuc_1101* (MUL2B, a PilM protein), and *amuc_1102* (MUL2A, structurally similar to an archaeal type IV pilin) (Fig. 2D). Among the 352 genomes within the family *Akkermansiaceae* that do not belong to *A. muciniphila*, 266 encoded *MUL2A* and *MUL2B*, and their *MUL2* loci exhibited conserved synteny in aquatic *Akkermansiaceae* (Fig. 2B, Fig. S3, Fig. S4A). We also found that the *MUL2* locus, though often conserved in other families of the order *Verrucomicrobiales*, was less conserved in order *Chthoniobacterales* and not detected in *Methylacidiphilales* (Fig. S4B). We hypothesize that the capacity for attachment to particles for substrate concentration is a common strategy among members of order *Verrucomicrobiales* and not just primarily found in the genus *Akkermansia* as previously thought [4, 14]. Even though the synteny was conserved, protein sequence identities of the putative *MUL2* locus components in environmental *Akkermansiaceae* genomes were low (bimodal distribution, ~20–100% identity, Fig. S5C–D). We compared the predicted structures of MUL2A to the experimentally obtained structure of amuc_1102 (protein 7DSZ, Fig. S5C–D, Fig. S6). MUL2A proteins were detected in 269 non-*A. muciniphila* aquatic and gut representative genomes (Fig. 2A, Fig. S5A–B, Fig. S6). As expected, the MUL2A structure predictions from gut-derived genomes were most similar to the crystal structure (*n* = 36, mean average local distance difference test (pLDDT) 83.6%*,* RMSD 0.66 Å (max. 3 Å), Fig. S5A–B). Marine (*n* = 133, mean pLDDT 88.5%, mean RMSD 1.07 Å (max. 3 Å)) and freshwater (*n* = 100, mean pLDDT 88.9%, mean RMSD 1.01 Å (max. 3 Å)) proteins displayed strong structural conservation (Fig. 2). The evaluation of MUL2B models was constrained by the absence of a reference structure, preventing direct comparison but highlighting it as a target for future structural characterization (Extended Results).

*Verrucomicrobiota* possess periplasmic regions that accumulate partially degraded glycans [15]. Aquatic *Pedosphaeraceae* store laminarin in the periplasm via selfish polysaccharide uptake [15, 16], whereas in *A. muciniphila* this process depends on *mul* genes [4]. Given the presence of *mul*-dependent attachment and transport systems and their capacity for FCSP degradation predicted above, aquatic *Akkermansiaceae* likely degrade complex glycans using a similar mechanism. Incubations of marine water with fluorescently labeled fucoidan (FLA-Fu) confirmed intracellular accumulation in *Akkermansiaceae* (Fig. 2C; Fig. S7; Fig. S8), providing experimental support for FCSP uptake and supporting uncovering selfish uptake as a phylogenetically conserved strategy for polysaccharide utilization across marine and gut *Verrucomicrobiota* [17, 18]. Although members of this lineage share a conserved glycan uptake and degradation framework, marine clades appear specialized for FCSPs, whereas the gut-associated *Akkermansia* species preferentially degrade mucin. Both substrates are fucosylated and sulfated glycans, suggesting that adaptation to the gut may have involved substrate-level specialization within an existing metabolic framework. This evolutionary continuity is consistent with the presence of an ancestral glycan degradation toolkit in *Akkermansia* [19] and suggest that this pre-existing framework may have enabled ecological flexibility across aquatic and host-associated environments.

We conclude that the ecological success of *Akkermansiaceae* likely relies on conserved mechanisms for attachment, transport, and intracellular degradation of complex glycans. Substrate-specific proteins fine-tune this system, enabling the utilization of glycans across environments.

## Supplementary Material

Akker_-_supplementary_-_figures_wrag096

Akker_-_tables_-_revision1_wrag096

Akker-manuscript-supplement-v3-revision1_wrag096

## Data Availability

The database generated in the current study is available in a GitLab repository, http://gitlab.mpi-bremen.de/ecological-genomics/akkermansiaceae-across-environments
