# Supplementary material for: Conserved glycan-utilization strategies shape Akkermansiaceae success across aquatic and gut ecosystems
Source: ISME J. Author manuscript; Available in PMC 2026 Jul 15. (PMC13174399; doi:10.1093/ismejo/wrag096)
Supplement: Supplementary figures [file EMS213909-supplement-Supplementary_figures.pdf]

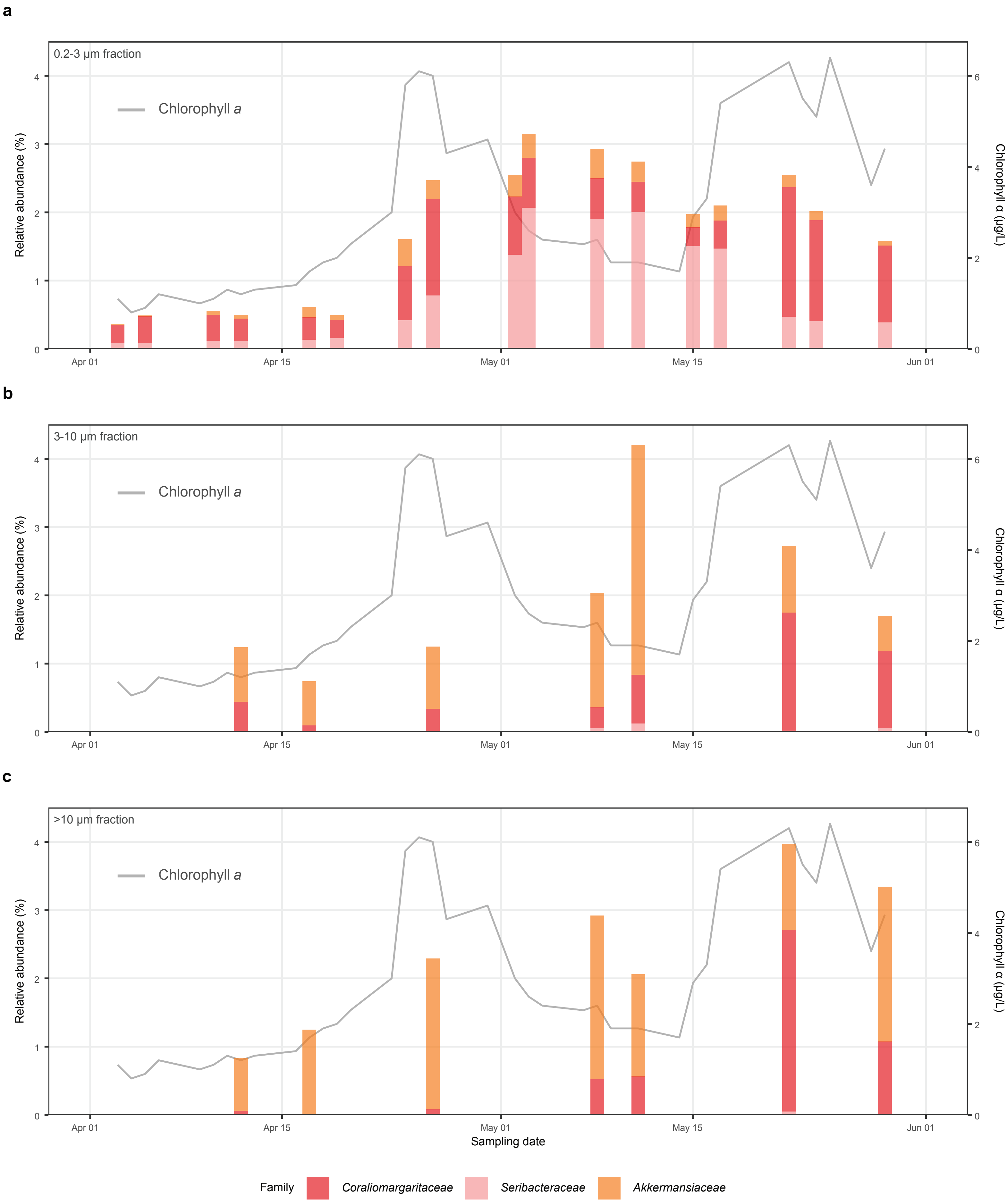

Figure S1. Relative abundances of *Verrucomicrobiota* MAGs belonging to families *Akkermansiaceae*, *Coralomargaritaceae*, and *Seribacteraceae* during the 2018 spring bloom within **a.** 0.2 - 3, **b.** 3 - 10, and **c.** >10 µm size fractions.

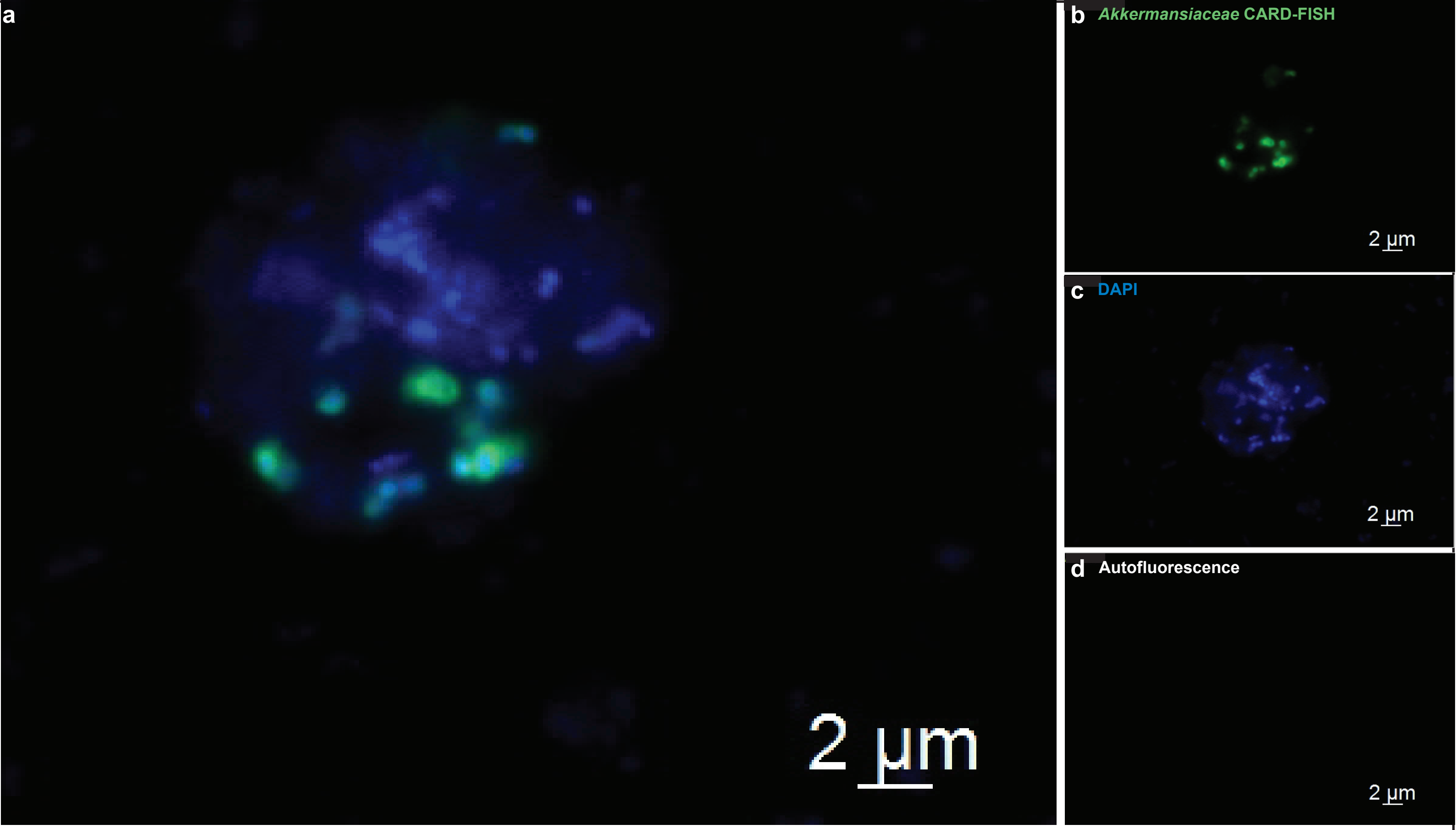

Figure S2. Epifluorescence photomicrograph of particle-attached *Akkermansiaceae*. **a.** A merged image showing CARD-FISH, DAPI, and autofluorescent signals. **b.** *Akkermansiaceae* CARD-FISH, **c.** DAPI, and **d.** autofluorescence channels are shown as individual channels. Scale bar shows 2 µm.

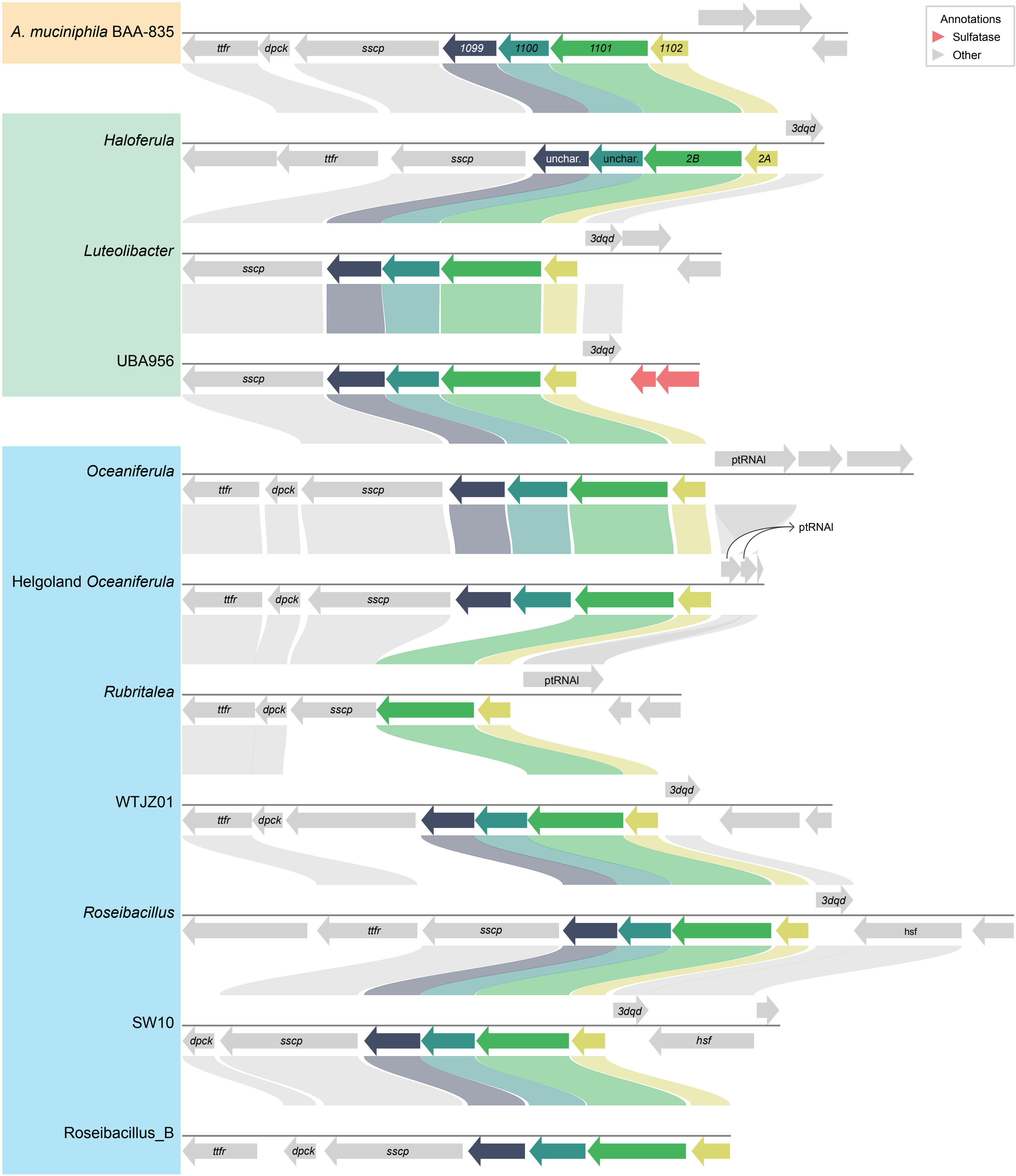

Figure S3. Synteny of the genus representatives MUL2 loci derived from gut, freshwater, and marine environments. The synteny of the MUL2 locus of the Helgoland-derived *Oceaniferula* genome is also included. Other conserved genes flanking the locus are labelled: secretion system channel protein (*sscp*), dephospho-CoA kinase (*dpck*), transcription termination factor Rho (*ttfr*), 3-dehydroquinate dehydratase (*3dqd*), proline tRNA ligase (ptRNAl), and helicase superfamily 1/2 (*hsf*).

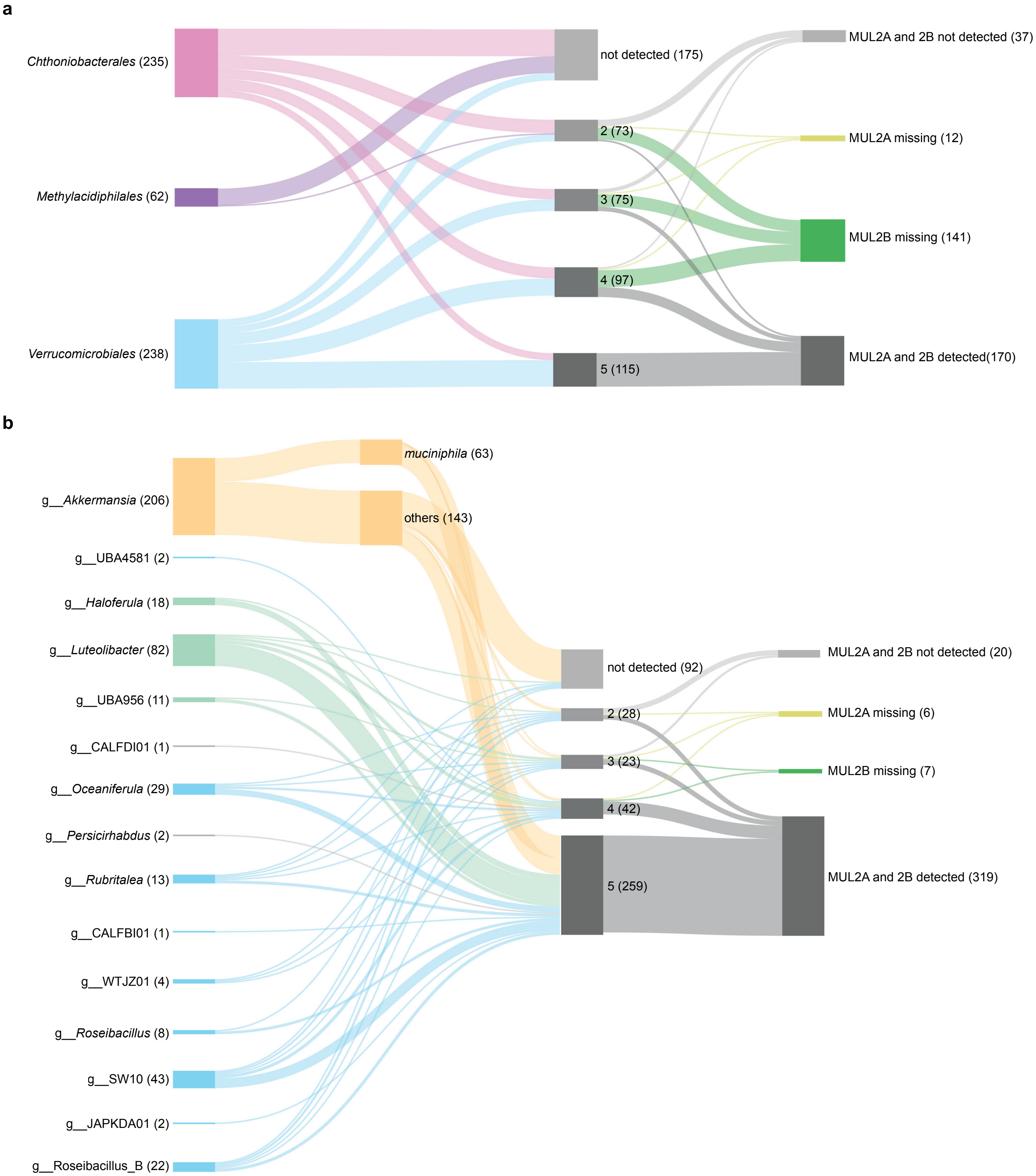

Figure S4. Conservation of the MUL2 locus within class *Verrucomicrobiia*. The prevalence of the MUL2 locus was examined based on the detection of type II/III secretion system protein (homolog of amuc_1098), two uncharacterized proteins (homologs of amuc_1099, amuc_1100, and amuc1102), and cell division protein *ftsA* (homolog of amuc_1101). **a.** Conservation of the *MUL2* locus across the class *Verrucomicrobiia*: orders *Chthoniobacterales*, *Methylacidiphilales*, and non-*Akkermansiaceae Verrucomicrobiales*. Within the order *Verrucomicrobiales*, the families DEV007, JBCCJK01, SLCJ01, V1-33, and *Verrucomicrobiaceae* are presented. From left to right, the nodes indicate: genus-level taxonomy and number of species within each genus in parentheses, the number of genes conserved in the locus (ranging from "not detected" to different numbers of genes detected), and whether MUL2A or MUL2B were not detected in the identified locus. **b.** Conservation of the *MUL2* locus across the family *Akkermansiaceae*. From left to right, the nodes indicate: genus-level taxonomy and number of species within each genus in parentheses, the number of genes conserved in the locus (ranging from "not detected" to different numbers of genes detected), and whether MUL2A or MUL2B were not detected in the identified locus.

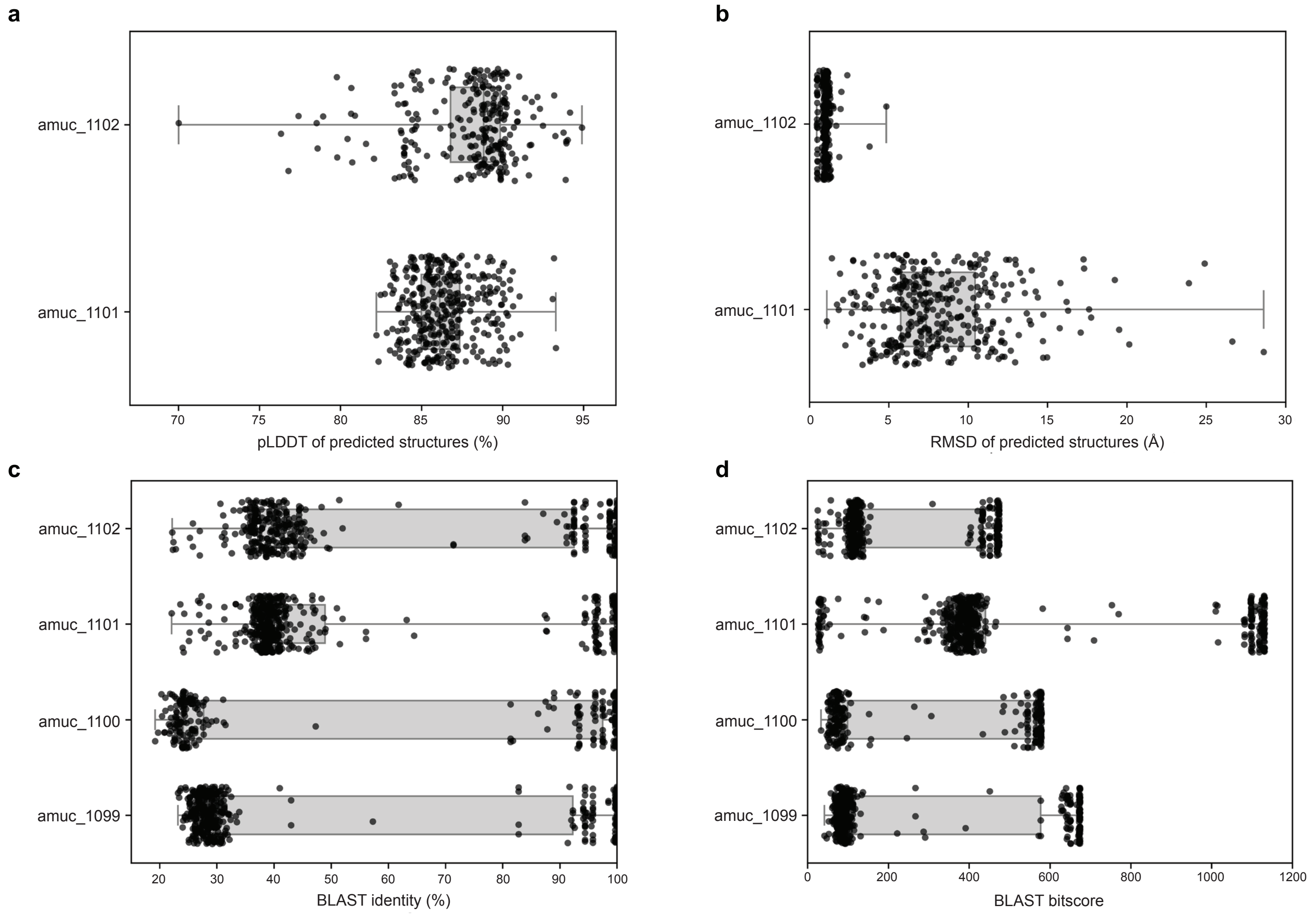

Figure S5. Similarity between MUL2 components from *A. muciniphila* and environmental sequence homologs. **a.** pLDDT values from MUL2A and MUL2B protein structure predictions. **b.** RMSD of all MUL2A and MUL2B structure predictions against the 7DSZ experimental reference (for amuc_1102) and the MUL2B in silico structure modeled from *A. muciniphila* . **c.** Sequence identities between five MUL2 locus protein sequences and predicted homologs in environmental genomes. **d.** Bitscore values derived from the sequence comparisons in c.

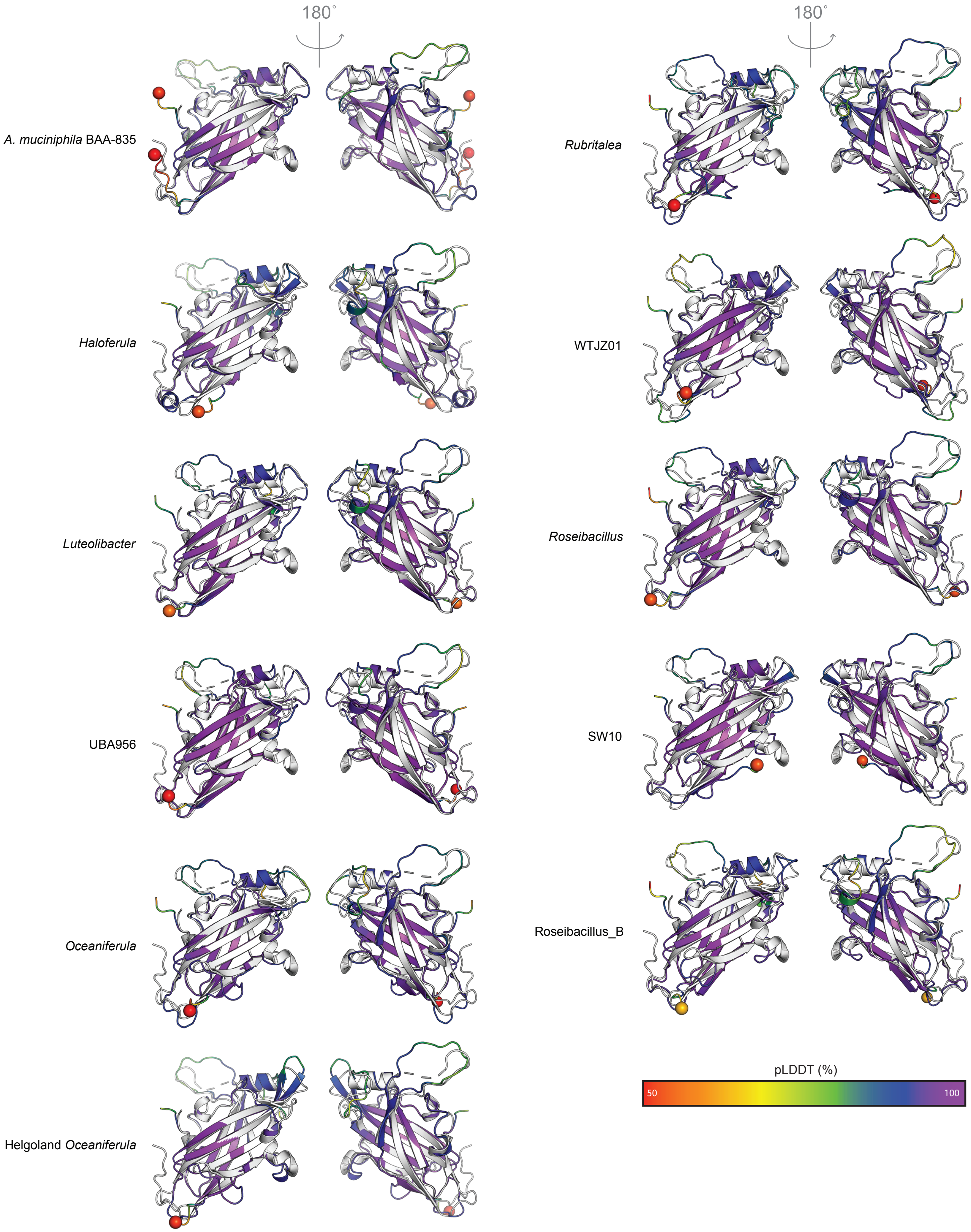

Figure S6. MUL2A predicted structures for each genus-representative genome superimposed onto the experimentally obtained MUL2A structure (7DSZ, in white). Protein structures are shown from the front and the back and are colored by pLDDT. The scales range from 50% (red) to 100% (purple). Signal peptides were removed and are depicted as spheres in the predicted structures.

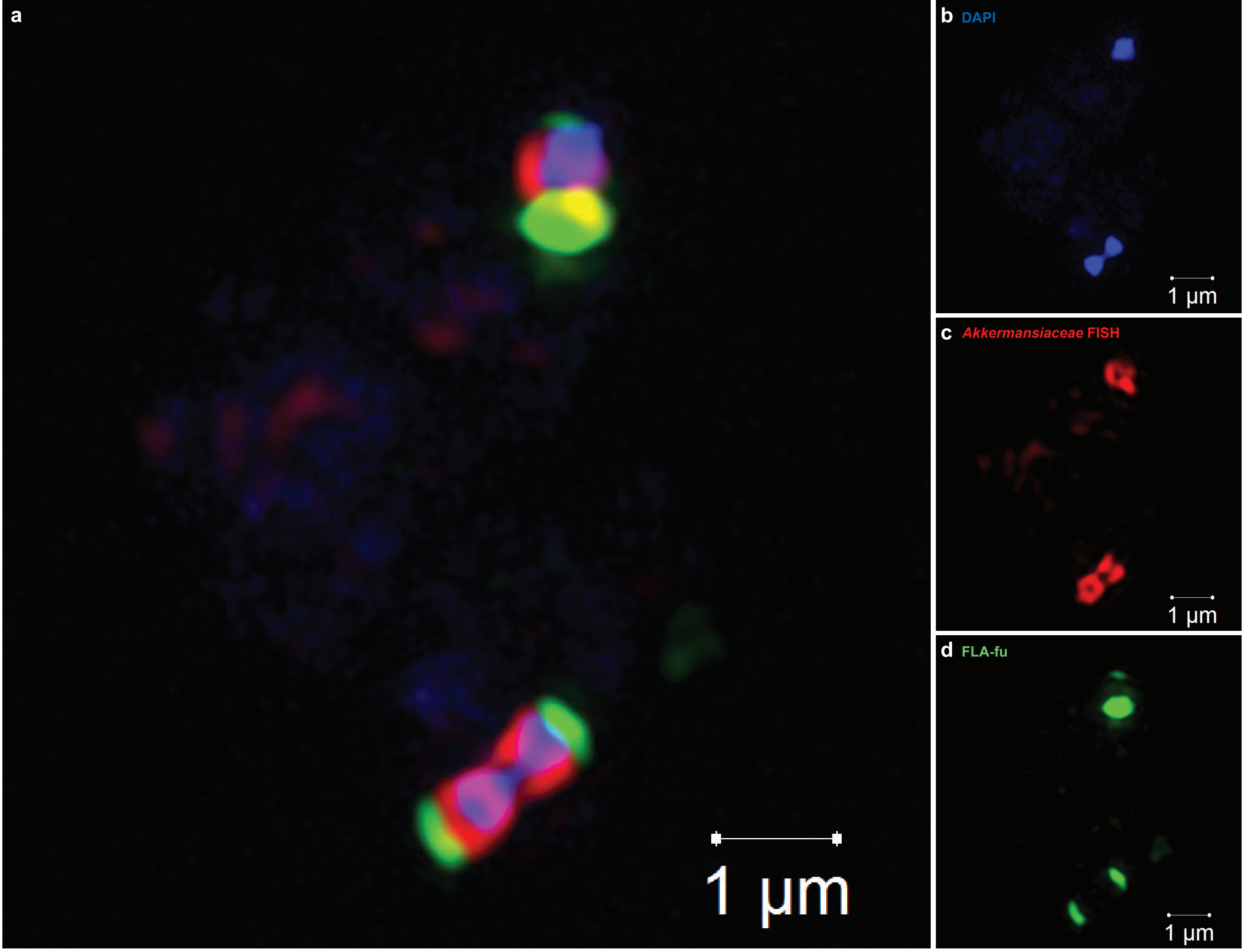

Figure S7. Super Resolution-Structural Illumination Microscopy (SR-SIM) of *Akkermansiaceae* cells showing fluorescently labeled fucoidan uptake. **a.** Merged image showing all channels superimposed. **b.** image of cells (DAPI, blue). **c.** CARD-FISH labeled *Akkermansiaceae* (red). **d.** Fluorescently labeled fucoidan uptake (FLA-fu, green).

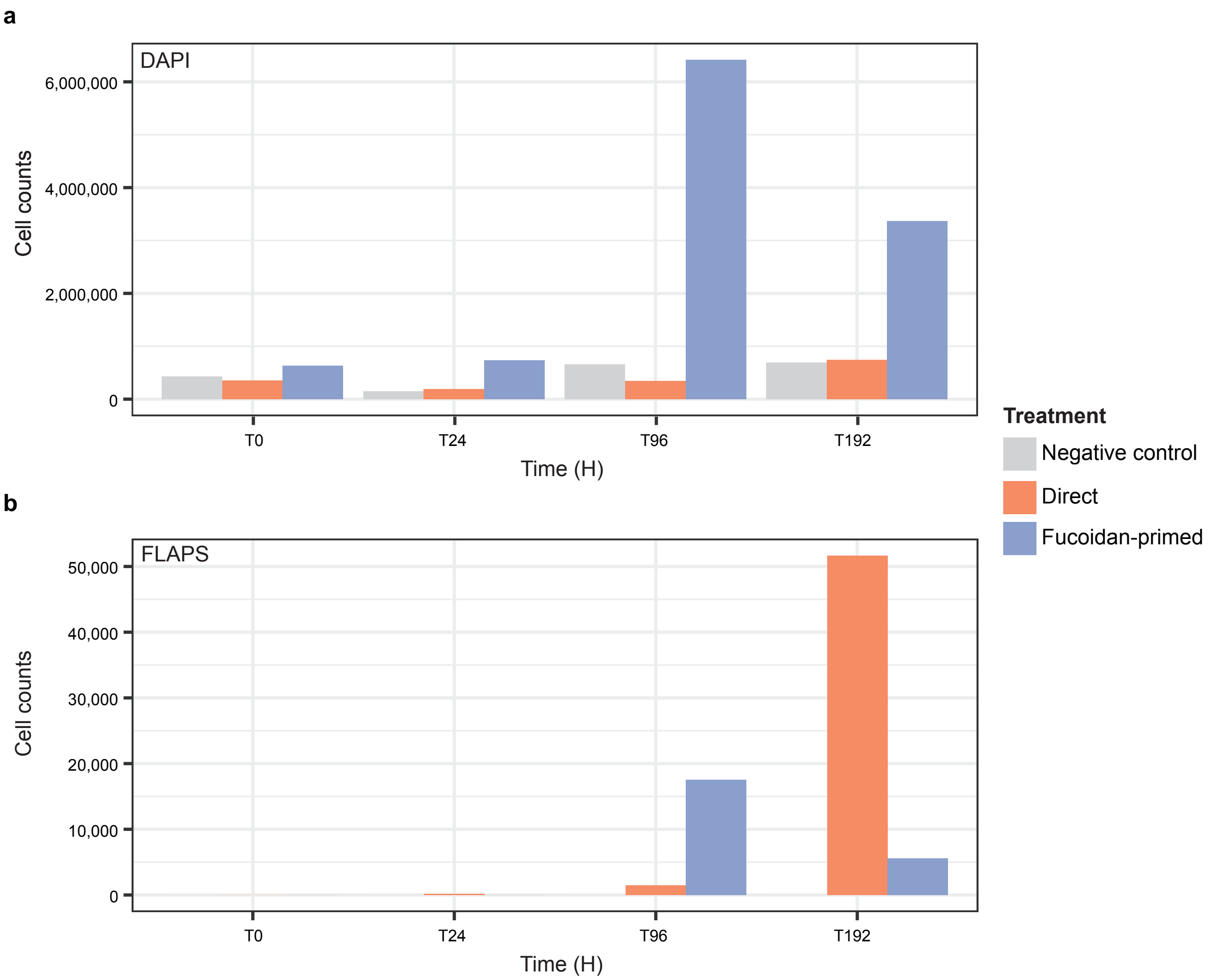

Fig S8. DAPI **a.** and FLA-Fuc **b.** cell counts over the course of FLA-Fuc incubations. Timepoints are given in hours, up to 8 days. Cell counts are shown for untreated seawater (negative control, gray), incubations with direct FLA-Fuc addition (orange), and for incubations which were primed with fucoidan prior to FLA-Fuc addition (blue).

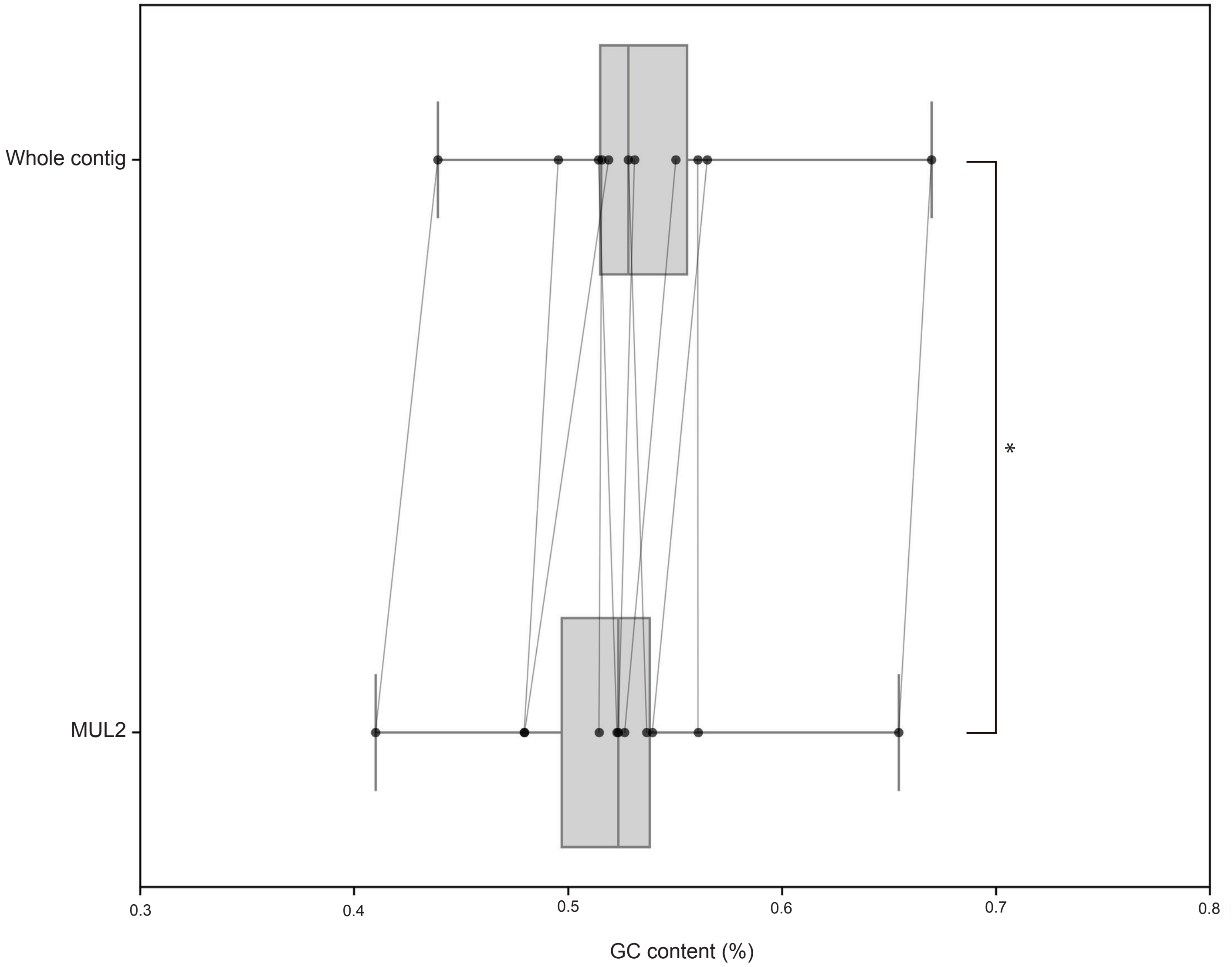

Figure S9. GC content comparison between each predicted MUL locus and the rest of the contig in which the locus was predicted. Lines denote paired points from the same MAG. Wilcoxon signed-rank test p-values < 0.05 for the pairs are indicated by *.
