# Supplementary material for: Conserved glycan-utilization strategies shape Akkermansiaceae success across aquatic and gut ecosystems
Source: ISME J. Author manuscript; Available in PMC 2026 Jul 15. (PMC13174399; doi:10.1093/ismejo/wrag096)
Supplement: Supplementary material [file EMS213909-supplement-Supplementary_material.pdf]

Methods

Genomes and metagenome-assembled genome collection

All genomes were recovered from publicly available datasets. The Genome Updater script [1] was used to download all genomes assigned to the family *Akkermansiaceae* according to GTDB r220. Additional genomes were downloaded based on publications assessing the microbial communities of algae and coral-associated environments [2–7]. GTDB-Tk v2.4.0 [8, 9] classify workflow was used to determine taxonomic affiliation. All collected genomes were filtered by quality as determined in checkM v1.2.3 [10] (completeness - 5x redundancy >=70), and dereplicated using dRep v3.4.5 [11] with a secondary average nucleotide identity threshold of 99% to reduce redundancy. Representative genomes were identified for *Akkermansiaceae* genera containing more than four genomes using dRep based on quality scores. Quality score was computed as: completeness - (5 x redundancy) + log(N50). We used Sandpiper v0.3.0 [12, 13] to determine the presence of members of the family *Akkermansiaceae* in metagenomic datasets from different environments.

One *Luteolibacter* genome (GCA_026397725) had a genome size of 12.6 Mb (Table S1), larger than the genus average of 3.64 Mb. As manual refinement using anvi'o v8 [14–18] did not suggest explicit contamination (4.2% redundancy and 95.8% completeness), and taxonomy estimates based on single-copy genes only indicated affiliation with *Luteolibacter*, we opted to keep the genome for further analyses. For visualization purposes, the genome size scale in Fig. 1A was capped at 8 Mb, corresponding to the second-largest genome size in the dataset.

Phylogenetic reconstructions

The alignment of 120 conserved genes for the selected genomes was generated in GTDB-Tk. A maximum likelihood tree was constructed using IQ-TREE v2.0.3 [19] with 100 bootstraps [20]

and the ModelFinder Plus parameter (-B 1000 -m MFP) [21], which selected the substitution model of LG+F+R10. This tree was then visualised using the interactive Tree of Life (iToL) [22]. The topology of the phylogenetic tree was verified using a partitioned alignment of 16 ribosomal proteins as detailed in Hug et al. 2016 and Hug et al. 2013. The ribosomal proteins were detected for all proteins in each genome using a python v3 compatible version of the IdentifyHMM script [23]. The detected protein sequences of interest were then aligned using muscle v5.1 [24], and trimmed with trimAl v1.5.rev0 build[2024-05-27] [25], both with default settings. The trimmed alignments were concatenated using Aln.cat.rb [26], and IQ-TREE was used to construct the tree with the settings above, including the coordinates file for the partitioned alignment [27].

Metabolic predictions and functional annotations

Bacterial microcompartments (BMCs) were detected using hmmsearch (HMMER v3.2.1 [28]) and a BMC domain model (PF00936; trusted cutoff). To reduce false-positive annotations, carbohydrate-active enzymes (CAZymes) were detected using two approaches, and only proteins detected in both methods were considered [29]. A search using hmmscan against the dbCAN v12 [30] followed by DIAMOND BLASTp v2.1.4.158 [16, 31] searches against the 2023 CAZy database [29]. Best matches were identified using BlastTab.best_hit_sorted.pl [26], and later based on a minimum 40% identity and an alignment length ≥ 50% of the query sequence. Sulfatases were detected using DIAMOND BLASTp against the SulfAtlas v2.3.1 database [32, 33], whereas peptidases were annotated against the MEROPS v124 database [34]. Matches derived from SulfAtlas and MEROPS were filtered using the same identity and alignment length indicated above.

Identifying candidate MUL proteins, structural prediction, and synteny analyses

Proteins were predicted across all representative genomes using PRODIGAL v2.6.3 [14]. Candidate MUL proteins in *Akkermansiaceae* genomes were identified using DIAMOND BLASTp v2.0.15.153 against the MUL protein sequences as detailed in [35]. Best hits were identified as described above, and post-search filters were applied to select for hits with at least 50% alignment length and an e-value ≤ 0.01. Next, protein structures for selected matches were predicted. For sequences shorter than 400bp, structures were predicted using ESMfold as built into the FoldSeek search server [36]. Longer sequences were first run through AlphaFold2 v2.3.2 [37] for structure prediction using the default settings and databases, and then queried using FoldSeek. All FoldSeek searches were performed against the PDB100 database, to identify homologous structures that were obtained and verified experimentally. For the synteny analysis, proteins neighbouring the candidate MUL sequences were annotated using InterProScan [38]. The synteny of confirmed MUL candidates was then examined using plots generated by pyGenomeViz v1.4.1 [39].

A different strategy that reduced computational time was implemented for detecting MUL structural homologs in non-*Akkermansiaceae* genomes. Structures of all candidate MUL2A and MULB sequences that resulted from a sequence identity search (as implemented above) were predicted in AlphaFold2. An average local distance difference test (pLDDT) filter of at least 70% confidence was applied to all structures. This cutoff ensures the selection of predicted structures ranging from well-modelled to highly accurate [40]. For the analyses of MUL2A, the predicted structures were individually superimposed to an experimentally characterized amuc_1102 structure (7DSZ [41]). The root mean square deviation (RMSD) between predicted structures and the experimental reference was calculated in PyMol v3.0.0 [42]. RMSD values were compared and interpreted in relation to the genome source. Because no experimentally resolved structure is available for MUL2B, we used an in silico predicted structure of MUL2B

(amuc_1101) from a genome in our dataset (GCF_028994115.1_ASM2899411v1), which shares 100% ANI with *A. muciniphila* strain BAA-835, for consistency with previous analyses of MUL2A.

To examine the conservation of the *MUL2* locus outside family *Akkermansiaceae* , we used species representatives for orders *Chthoniobacterales*, *Methylacidiphilales, and Verrucomicrobiales*. Within the order *Verrucomicrobiales*, species representatives of the following non-*Akkermansiaceae* families were utilized: EV007, JBCCJK01, SLCJ01, V1-33, and *Verrucomicrobiaceae*. Altogether, a total of 535 genomes were selected. Genomes were annotated using InterProScan v5.75-106.0 with default settings, and the detection of homologs for *amuc_1098 - amuc_1102* were examined in each genome. To ensure consistent results, the same pipeline was repeated using the environmental *Akkermansiaceae*.

Visualization of particle attached cells in surface seawater

Surface seawater samples were collected from the 'Kabeltonne' ecological research site off the North Sea island of Helgoland (54˚ 11.3′ N, 7˚ 54.0′ E), as described previously [43]. Collected seawater for cell counting and visualization was fixed by adding formaldehyde (final concentration of 1%) directly to the sample, followed by filtration on 0.2 µm polycarbonate filters without pre-filtration [44].

For catalyzed reporter deposition-fluorescence *in situ* hybridization (CARD-FISH) analysis, we chose samples from 2018 collected on April 24, May 3, May 8, May 11, and May 15 based on relative abundance results of metagenomic analyses. We used the oligonucleotide probe EUB338-III to target the order *Verrucomicrobiales [45]*. In addition, we used three clade-specific probes, c7-182, c13-1000, and c21-472, that target members of the *Verrucomicrobiota* families *Akkermansiaceae*, *Coraliomargaritaceae* (previously DSM-45221), and *Seribacteraceae*

(previously MB11C04 [46]), respectively [47]. Lastly, we used oligonucleotide probes EUB338-I-III and NON338 as positive and negative controls [48, 49] (Table S2).

We performed CARD-FISH as previously reported [50]. First, the filters were cut into smaller sections and embedded in 0.1% (w/v) low-gelling-point agarose (Biozym) for 20 min at room temperature (RT). Then, the filter sections were incubated in lysozyme solution (10 mg/mL; buffer: 0.05 M EDTA pH 8, 0.1 M Tris-HCl, pH 8) for one hour at 37°C to permeabilize the cell walls. The filter sections were incubated in 10 mL 0.15% H_2O_2/Methanol (MeOH) solution for 30 min at RT to inactivate the endogenous peroxidases. All hybridizations were done in hybridization chambers for two hours at 46°C, with a 300:1 ratio of hybridization buffer and 8.4 pmol/µL probe. Hybridization chambers were prepared by stacking Petri dishes in a plastic container. Then, a tissue paper soaked with a mixture of 2.25 M NaCl and 5 mL of formamide, as required by the probe (Table S2), was placed at the bottom of the container. After hybridization, the filter sections were washed using washing buffer (buffer: NaCl (depending on the formamide concentration as required by the probe, Table S2), 5 mM M EDTA pH 8, 20 mM Tris-HCl, pH 8, 0.01% SDS, and sterile MilliQ water) for 15 min at 48°C. The filter sections were incubated in 1X phosphate-buffered saline (PBS) for 15 min at RT for the amplification step. Then, these were incubated with a detection solution (1,000 µL amplification buffer, 10 µL 0.15% H_2O_2-1X PBS, 1 µL Alexa-488 tyramides) for 45 min at 46°C in the dark. The filter sections were rinsed with 1X PBS for 10 min at RT in the dark and then with 96% ethanol for 1 min. Afterward, the filter sections were left to air-dry on Whatmann paper. Lastly, the filter sections were mounted on clean glass slides with two drops of mounting medium (1 mL Citifluor with two drops of NucBlue).

DAPI (4',6-diamidino-2-phenylindole)-positive and probe-positive cells were visualized and counted using an epifluorescence microscope Axioimager.Z2m microscope (Zeiss, Jena, Germany) and Automated Cell Measuring and Enumeration Tool (ACMEtool) v2021-04-02 [51].

Particle-attached cells were visualized using a confocal laser scanning microscope LSM 780 (Zeiss, Jena, Germany), with 488 and 405 nm lasers. Z-stack images were acquired with a PlanApochromat 63x/1.4 Oil objective and processed in Zen Black (Carl Zeiss Microscopy, Germany). Absolute counts for *Verrucomicrobiota* cells are given in Table S3, as well as absolute counts for *Verrucomicrobiota* families *Akkermansiaceae*, *Coraliomargaritaceae*, and *Seribacteraceae*.

Visualisation of fluorescently labelled fucoidan uptake

For fluorescent-substrate incubations, surface seawater samples were collected on 3. May 2023 at the long-term ecological research station “Kabeltonne”, as described previously. Incubations were performed using fluorescently labelled fucoidan (FLA-Fuc), or a mixture of unlabelled fucoidan and FLA-Fuc. Both fucoidans were derived from *Fucus vesiculosus* (Sigma-Aldrich Chemie GmbH, Munich, Germany). The FLA-Fuc was fluorescently labelled with fluoresceinamine (Sigma-Aldrich; isomer II) as described by [52]. Free monosaccharides and oligosaccharides were removed by repeated size exclusion chromatography, ensuring the removal of low molecular weight carbohydrates. Incubations were performed in duplicate for two treatments. In the first (“Direct FLA-Fuc”), 3.5 µM FLA-Fuc was added to the water sample before the incubation (Time point 0). In the second (“Fucoidan-primed”), 500 mL water samples received 3.5 µM unlabelled fucoidan at time 0, then 35 µM FLA-Fuc at Time 72). A 500-mL water sample without substrate addition served as the negative control.

For every time point, a 10 mL subsample was fixed with sterile formaldehyde (final concentration 2%) for 1h at RT and subsequently filtered onto a 47 mm 0.2 µm polycarbonate filter with a 0.45 µm support filter (GTTP, Millipore, Eschborn, Germany), applying a gentle vacuum of -<200 mbar. Fluorescence *in situ* hybridization (FISH) was performed as described by [53]. For cell enumeration, we used 0.83 pmol μL^{-1} EUB338-III, and for visualization, we used

0.83 pmol μL^{-1} 4xAtto594-labelled RUB390 probe (see Table S4). The hybridization buffer was composed of 900 mM NaCl, 20 mM Tris-HCl (pH 7.5), 0.02% sodium dodecyl sulphate, 10% dextran sulphate (wt/vol) and 1% (wt/vol) blocking reagent (Boehringer; Mannheim, Germany), and the formamide concentration of 20%. Hybridizations were carried out at 46 °C in a humidity chamber for 2 hours, with a subsequent wash in a buffer containing 80 mM NaCl, 20 mM Tris/HCl (pH 8), and 0.01% sodium dodecyl sulfate at 48 °C. Filter sections were removed from the wash buffer, air-dried on Whatman paper and mounted on 4:1 CitiFluorAF1 (CitiFluor Ltd., London, England) and Vectashield (Vector Laboratories, Burlingame, California, USA) containing 1 μg ml^{-1} DAPI (4′,6-diamidino-2-phenylindole; Sigma-Aldrich, Steinheim, Germany).

The FISH signals and FLA-Fuc uptake were initially visualized by epifluorescence microscopy (Nikon Eclipse 50i) and then enumerated by a fully automated microscope imaging system, described in detail by [51] on a AxioImager.Z2 microscope stand (Carl Zeiss MicroImaging GmbH, Göttingen, Germany). Subsequently, the images were imported into the ACMETOOL3.0 (MPI Bremen) image analysis software for the cell counts. Total DAPI cell counts are given in Table S5, as well as counts for cells that took up FLA-Fuc. Representative images demonstrating the FLA-Fuc uptake of the *Verrucomicrobiota* cells were acquired using Laser Scanning Microscope (LSM780, Zeiss, Jena, Germany), equipped with an Airyscan detector and Super Resolution-Structured Illumination Microscopy with intensity line profiles of individual cells generated in ZEN black software (Carl Zeiss).

Metagenome-assembled genome abundances in the North Sea

Samples recovered during the spring bloom of 2018 in North Sea (see above) were used for the examination of *Akkermansiaceae* MAGs in different size fractions (samples were pre-filtered using >10 and 3μm pore-size polycarbonate filters, and free-living cells were collected on 0.2μm filters). The short-read metagenomic samples were previously quality-filtered, assembled, and

used for metagenome-assembled genome recovery [54]. Medium-to-high quality MAGs (Q = Completeness(%) - (5 * Redundancy(%)) ≥ 50) were selected and taxonomy was determined using GTDB-Tk v2.4.0 with r220 (default parameters).

Representative MAGs for the phylum *Verrucomicrobiota* were determined with dRep v3.5.0 using Mash v1.1.1 [55] with a threshold of 90% ANI for primary clustering, and fastANI v1.33 [56] with a threshold of 97% ANI and a minimum overlap between genomes of 0.25 for secondary comparisons. To determine MAG abundances, metagenomic reads were competitively mapped to the MAGs using Minimap2 v2.28-r1209 [57] (-sr). The trimmed mean coverage (i.e., sequencing depth or mean coverage of the contigs after removing the 5% of bases with the highest and lowest coverage) was calculated using CoverM v0.7.0 [58]. These values were normalized by the genome equivalents obtained from MicrobeCensus v1.1.1 [59] as reported before [46].

Extended Results

Previous analyses of North Sea *Verrucomicrobiota* proposed the genus *Candidatus* Mariakkermansia, with type species *Candidatus* Mariakkermansia forsetii (GCA_964187645; 20100303_Bin_52_1) [47]. This genome was incorporated into GTDB in release R226, after the compilation of the 444-genome dataset analyzed here. The type material genome exhibits 99.2% ANI to genome GCA_002470485 in our dataset, which is classified as *Oceaniferula* in the latest release of GTDB.

Clusters of genes encoding the detection, uptake, and degradation of carbohydrates are classified as polysaccharide utilisation loci (PULs) and are typically found in members of the phylum *Bacteroidota* [60, 61]. In addition, some of the genes found in PULs typically contain

carbohydrate-binding domains. Nonetheless, within the MUL2 loci detected here and the surrounding genes we were unable to identify genes with carbohydrate binding motifs.

Conserved synteny of the MUL1 locus was not detected in aquatic *Akkermansiaceae* genomes. Additionally, no homologs for MUL1B (amuc_0543) were detected in aquatic genomes. Without annotations indicating MUL1B homologs , it was not possible to predict a locus based on other expected components of MUL1, such as ExbD/B, which were detected throughout the genomes. Furthermore, no experimentally verified *MUL1* crystal structures were available during the analyses presented here, thus limiting a structural protein-search approach. Therefore, the limited detection of homologues for the components of MUL1 likely indicates that *Mul1B* genes are gut-specific in *A. muciniphila*, and that other aquatic species might encode host- or environment-specific genes.

Amino acid sequence-based analyses of the predicted *MUL2* locus in aquatic *Akkermansiaceae* mainly resulted in matches to sequences derived from *A. muciniphila* strains [35] and other family members. The amino acid identities resulting from the protein sequence searches ranged from ~20 to 100%. Specifically, the identities of the homologous uncharacterized proteins (amuc_1099 and amuc_1100) ranged from 23.2% to 100% and 19.2% to 100%, respectively (Fig. S5c-d). Both searches exhibited a largely bimodal distribution of identity, with most hits between 20% and 40% and between 80% and 100%. This broadly bimodal distribution was not identified for MUL2A and MUL2B homologs (identities for both proteins ranged from 22.2% to 100%, Fig. S5c-d).

As an experimental protein structure has not been determined for MUL2B (*amuc_1101*), thus, we conducted structural comparisons using a predicted MUL2B derived from *A. muciniphila* (strain BAA-835, GCF_028994115). This predicted structure has a pLDDT of 83.9% (thus within the well-modeled range [40]). When comparing the predicted structures from the

Akkermansiaceae dataset to this reference, 15 genomes had predicted MUL2B proteins with average pLDDT > 70% and RMSD < 3Å compared to the *A. muciniphila* predicted structure (Fig. S5a-b). These were mostly derived from genomes recovered from gut environments (n=13, average pLDDT 84.76%, average RMSD 2.41 Å), though two were predicted from marine genomes (average pLDDT 92.2%, average RMSD 2.47 Å). Additionally, 339 predicted structures exhibited pLDDT > 70%, yet had RMSD > 3 Å to the predicted MUL2B structure of *A. muciniphila* (average RMSD 8.50Å, ranging from 3.20 - 28.61 Å; average pLDDT 86.4%, ranging from 82.2 - 93.2%). While an RMSD value below 3Å can indicate that two structures are similar, a distance above 3Å is not recommended to be used on its own to indicate structural similarity. The analyses presented here should be validated once an experimental structure of *amuc_1101* is obtained. Compared to the analysis of MUL2A (see main results section), the predicted structures of MUL2B had a smaller range of pLDDT values (pLDDT values from 82.2% to 93.3% compared to amuc_1102 ranging from 70.0% to 94.9%). Nonetheless, MUL2B comparisons revealed a larger distance when superimposed on the *in silico* predicted structure reference (RMSD for MUL2B comparisons ranged from 1.09 Å to 28.61 Å, compared to values between 0.46 Å and 4.84 Å for MUL2A). While this difference in the distributions of MUL2A and MUL2B observations could be due to the use of different references (experimental for MUL2A versus predicted for MUL2B), it also suggests that MUL2A is more conserved, indicating its importance for the function of the MUL mechanism. Finally, *in silico* structural analyses of candidate amuc_1102 predicted structures of representative genomes revealed a signal peptide in predicted amuc_1102 structures, which were the lowest confidence regions of the predictions (Table S6, Fig. S6).

To detect possible conserved genetic features across gut and aquatic MUL2 loci, we further characterized these regions in representative genomes. In representative genomes, the GC content of the *MUL2* locus was lower compared to the rest of the contig harboring the predicted

MUL2 region (Fig. S9). Surrounding the *MUL2* regions in *Akkermansiaceae* genomes, we identified secretion system channel proteins, transcription termination factor Rho, dephospho-CoA kinases, tRNA ligases and synthases, among others (Fig. S3). Furthermore, we identified a conserved set of genes upstream of MUL2, including transcription factor Rho, dephospho-CoA kinase, and secretion system channel proteins (e.g., the genus representatives for *A. muciniphila*, *Oceaniferula*, *Rubritalea*, and the Helgoland *Oceaniferula* genome, Fig. S3). We also identified a suffix in the *Oceaniferula* genus representative and the Helgoland *Oceaniferula* genome, which includes tRNA ligases and a tRNA synthase. Other genomes instead have a 3-dehydroquinate dehydratase after the predicted *MUL2* region (e.g. the *Halorferula* and WTJZ01 genus representatives). Even though the *Rubritalea* genus representative lacks the entire *MUL2* locus (the two uncharacterized *amuc_1099* and *amuc_1100* genes were not identified in proximity to the *amuc_1101* and *amuc_1102* genes), it encodes the conserved set of upstream genes observed in the other genomes.

The analysis of the conservation of the *MUL2* locus based on the presence of multiple genes (*amuc_1098* - *amuc_1102*) revealed the taxonomic association of this mechanism within *Akkermansiaceae* (Fig. S4a and b). In most *A. muciniphila* genomes, all genes were identified in a locus (n=60/63 genomes). In other non-*A. muciniphila Akkermansia* species, the locus was mostly not conserved (n=86/143) (Fig. S4a). Nonetheless, the locus was conserved across all other genera within the *Akkermansiaceae* (n=212/238 genomes had ≥3 genes in the locus). Overall, about half of the genomes in the dataset had homologues to all five genes of the locus (n=249/444). Regarding the presence of *Mul2A* or *Mul2B*, both were detected in most genomes where the locus was identified (n=319/352). In some cases where the locus was less conserved, with two or three other genes identified, both were missing (n=20), and there were few instances where only one was missing (n=7 for *Mul2B*, n=6 for *Mul2A*). Based on these results, we conclude that the MUL2 locus is conserved across members of the

Akkermansiaceae family. A broader analysis including genomes phylogenetically closer to *Akkermansiaceae* within the same class, revealed that in most non-*Akkermansiaceae* members of the order *Verrucomicrobiales*, the MUL2 locus was also identified (n=190/238 genomes had ≥ 3 genes in the locus, Fig. S4b). This locus was detected in a smaller fraction of order *Chthoniobacterales* genomes (n=97/235 genomes had ≥ 3 genes in the locus). Moreover, we did not detect a strong presence of the MUL2 locus in order *Methylacidiphilales* genomes: only two genomes had two genes in the locus, and the locus was not identified in the other 58). Thus, we hypothesize that the *MUL2* locus is conserved in the class *Verrucomicrobiales*, and potentially in the *Chthoniobacterales*, however not in the class *Methylacidiphilales*, and therefore not conserved in all members of class *Verrucomicrobiia*.

Ultimately, these results highlight that we can detect partial conservation of the *MUL* loci, specifically *MUL2*, within groups related to family *Akkermansiaceae*. We hypothesize that a conserved mechanism might play a central role in the attachment to substrates to concentrate them near the cell surface for transport and degradation, which we identify as being central to the family *Akkermansiaceae,* regardless of their genomic source.

References

1. Piro VC. genome_updater. Github.
2. Song W, Wemheuer B, Steinberg PD, Marzinelli EM, Thomas T. Contribution of horizontal gene transfer to the functionality of microbial biofilm on a macroalgae. *ISME J* 2021; **15**: 807–817.
3. Weigel BL, Miranda KK, Fogarty EC, Watson AR, Pfister CA. Functional insights into the kelp microbiome from metagenome-assembled genomes. *mSystems* 2022; **7**: e0142221.
4. Miranda K, Weigel BL, Fogarty EC, Veseli IA, Giblin AE, Eren AM, et al. The diversity and functional capacity of microbes associated with coastal macrophytes. *mSystems* 2022; **7**:

e0059222.

5. Lu D-C, Wang F-Q, Amann RI, Teeling H, Du Z-J. Epiphytic common core bacteria in the microbiomes of co-located green (*Ulva*), brown (*Saccharina*) and red (*Grateloupia*, *Gelidium*) macroalgae. *Microbiome* 2023; **11**: 126.
6. Xu Y, Schultz-Johansen M, Yao H, Wilkie I, Klau LJ, Chen Y, et al. Phosphate starvation stops bacteria digesting algal fucan that sequesters carbon. *bioRxiv* . 2024. , 2024.04.07.588495
7. Zhang Y-S, Zhang Y-Q, Zhao X-M, Liu X-L, Qin Q-L, Liu N-H, et al. Metagenomic insights into the dynamic degradation of brown algal polysaccharides by kelp-associated microbiota. *Appl Environ Microbiol* 2024; **90**: e0202523.
8. Chaumeil P-A, Mussig AJ, Hugenholtz P, Parks DH. GTDB-Tk v2: memory friendly classification with the genome taxonomy database. *Bioinformatics* 2022; **38**: 5315–5316.
9. Chaumeil P-A, Mussig AJ, Hugenholtz P, Parks DH. GTDB-Tk: a toolkit to classify genomes with the Genome Taxonomy Database. *Bioinformatics* 2020; **36**: 1925–1927.
10. Parks DH, Imelfort M, Skennerton CT, Hugenholtz P, Tyson GW. CheckM: assessing the quality of microbial genomes recovered from isolates, single cells, and metagenomes. *Genome Res* 2015; **25**: 1043–1055.
11. Olm MR, Brown CT, Brooks B, Banfield JF. dRep: a tool for fast and accurate genomic comparisons that enables improved genome recovery from metagenomes through de-replication. *ISME J* 2017; **11**: 2864–2868.
12. Woodcroft BJ, Aroney STN, Zhao R, Cunningham M, Mitchell JAM, Nurdiansyah R, et al. Comprehensive taxonomic identification of microbial species in metagenomic data using SingleM and Sandpiper. *Nat Biotechnol* 2025; 1–6.
13. Eisenhofer R, Alberdi A, Woodcroft BJ. Large-scale estimation of bacterial and archaeal DNA prevalence in metagenomes reveals biome-specific patterns. *bioRxiv* . 2024. , 2024.05.16.594470

14. Hyatt D, Chen G-L, LoCascio PF, Land ML, Larimer FW, Hauser LJ. Prodigal: prokaryotic gene recognition and translation initiation site identification. *BMC Bioinformatics* 2010; **11**: 119.

15. Parks DH, Chuvochina M, Waite DW, Rinke C, Skarshewski A, Chaumeil P-A, et al. A standardized bacterial taxonomy based on genome phylogeny substantially revises the tree of life. *Nat Biotechnol* 2018; **36**: 996–1004.

16. Buchfink B, Xie C, Huson DH. Fast and sensitive protein alignment using DIAMOND. *Nat Methods* 2015; **12**: 59–60.

17. Eren AM, Kiefl E, Shaiber A, Veseli I, Miller SE, Schechter MS, et al. Community-led, integrated, reproducible multi-omics with anvi'o. *Nat Microbiol* 2021; **6**: 3–6.

18. Eren AM, Esen ÖC, Quince C, Vineis JH, Morrison HG, Sogin ML, et al. Anvi'o: an advanced analysis and visualization platform for 'omics data. *PeerJ* 2015; **3**: e1319.

19. Minh BQ, Schmidt HA, Chernomor O, Schrempf D, Woodhams MD, Von Haeseler A, et al. IQ-TREE 2: New Models and Efficient Methods for Phylogenetic Inference in the Genomic Era. *Molecular Biology and Evolution* 2020; **37**: 1530–1534.

20. Hoang DT, Chernomor O, von Haeseler A, Minh BQ, Vinh LS. UFBoot2: Improving the ultrafast bootstrap approximation. *Mol Biol Evol* 2018; **35**: 518–522.

21. Kalyaanamoorthy S, Minh BQ, Wong TKF, von Haeseler A, Jermiin LS. ModelFinder: fast model selection for accurate phylogenetic estimates. *Nat Methods* 2017; **14**: 587–589.

22. Letunic I, Bork P. Interactive Tree of Life (iTOL) v6: recent updates to the phylogenetic tree display and annotation tool. *Nucleic Acids Res* 2024; **52**: W78–W82.

23. Graham ED, Heidelberg JF, Tully BJ. Potential for primary productivity in a globally-distributed bacterial phototroph. *ISME J* 2018; **12**: 1861–1866.

24. Edgar RC. Muscle5: High-accuracy alignment ensembles enable unbiased assessments of sequence homology and phylogeny. *Nat Commun* 2022; **13**: 6968.

25. Capella-Gutiérrez S, Silla-Martínez JM, Gabaldón T. trimAl: a tool for automated alignment

trimming in large-scale phylogenetic analyses. *Bioinformatics* 2009; **25**: 1972–1973.

26. Rodriguez-R LM, Konstantinidis KT. The enveomics collection: a toolbox for specialized analyses of microbial genomes and metagenomes. 2016. PeerJ Preprints.

27. Chernomor O, von Haeseler A, Minh BQ. Terrace aware data structure for phylogenomic inference from supermatrices. *Syst Biol* 2016; **65**: 997–1008.

28. Eddy SR. Accelerated Profile HMM Searches. *PLoS Comput Biol* 2011; **7**: e1002195.

29. Drula E, Garron M-L, Dogan S, Lombard V, Henrissat B, Terrapon N. The carbohydrate-active enzyme database: functions and literature. *Nucleic Acids Research* 2022; **50**: D571–D577.

30. Zheng J, Ge Q, Yan Y, Zhang X, Huang L, Yin Y. dbCAN3: automated carbohydrate-active enzyme and substrate annotation. *Nucleic Acids Res* 2023; **51**: W115–W121.

31. Buchfink B, Reuter K, Drost H-G. Sensitive protein alignments at tree-of-life scale using DIAMOND. *Nat Methods* 2021; **18**: 366–368.

32. Barbeyron T, Brillet-Guéguen L, Carré W, Carrière C, Caron C, Czjzek M, et al. Matching the Diversity of Sulfated Biomolecules: Creation of a Classification Database for Sulfatases Reflecting Their Substrate Specificity. *PLOS ONE* 2016; **11**: e0164846.

33. Stam M, Lelièvre P, Hoebeke M, Corre E, Barbeyron T, Michel G. SulfAtlas, the sulfatase database: state of the art and new developments. *Nucleic Acids Research* 2023; **51**: D647–D653.

34. Rawlings ND, Barrett AJ, Thomas PD, Huang X, Bateman A, Finn RD. The MEROPS database of proteolytic enzymes, their substrates and inhibitors in 2017 and a comparison with peptidases in the PANTHER database. *Nucleic Acids Res* 2018; **46**: D624–D632.

35. Davey LE, Malkus PN, Villa M, Dolat L, Holmes ZC, Letourneau J, et al. A genetic system for *Akkermansia muciniphila* reveals a role for mucin foraging in gut colonization and host sterol biosynthesis gene expression. *Nat Microbiol* 2023; **8**: 1450–1467.

36. van Kempen M, Kim SS, Tumescheit C, Mirdita M, Lee J, Gilchrist CLM, et al. Fast and

accurate protein structure search with Foldseek. *Nat Biotechnol* 2023; 1–4.

37. Jumper J, Evans R, Pritzel A, Green T, Figurnov M, Ronneberger O, et al. Highly accurate protein structure prediction with AlphaFold. *Nature* 2021; **596**: 583–589.
38. Jones P, Binns D, Chang HY, Fraser M, Li W, McAnulla C, et al. InterProScan 5: Genome-scale protein function classification. *Comput Appl Biosci* 2014; **30**: 1236–1240.
39. Shimoyama Y. pyGenomeViz: A genome visualization python package for comparative genomics. 2024.
40. Database EAP. AlphaFold FAQ. https://alphafold.ebi.ac.uk/faq. Accessed 6 Aug 2025.
41. Xiang R, Wang J, Xu W, Zhang M, Wang M. Amuc_1102 from *Akkermansia muciniphila* adopts an immunoglobulin-like fold related to archaeal type IV pilus. *Biochem Biophys Res Commun* 2021; **547**: 59–64.
42. Schrödinger, LLC. The PyMOL Molecular Graphics System, Version 1.8. 2015.
43. Teeling H, Fuchs BM, Becher D, Klockow C, Gardebrecht A, Bennke CM, et al. Substrate-Controlled Succession of Marine Bacterioplankton Populations Induced by a Phytoplankton Bloom. *Science* 2012; **336**: 608–611.
44. Francis TB, Krüger K, Fuchs BM, Teeling H, Amann RI. *Candidatus* Prosiliicoccus vernus, a spring phytoplankton bloom associated member of the *Flavobacteriaceae*. *Syst Appl Microbiol* 2019; **42**: 41–53.
45. Daims H, Brühl A, Amann R, Schleifer KH, Wagner M. The domain-specific probe EUB338 is insufficient for the detection of all Bacteria: development and evaluation of a more comprehensive probe set. *Syst Appl Microbiol* 1999; **22**: 434–444.
46. Wilkie I, Orellana LH. Elusive marine *Verrucomicrobiota*: Seasonally abundant members of the novel genera *Seribacter* and *Chordibacter* specialize in degrading sulfated glycans. *Syst Appl Microbiol* 2025; **48**: 126562.
47. Orellana LH, Francis TB, Ferraro M, Hehemann J-H, Fuchs BM, Amann RI. *Verrucomicrobiota* are specialist consumers of sulfated methyl pentoses during diatom

blooms. *ISME J* 2022; **16**: 630–641.

48. Amann RI, Krumholz L, Stahl DA. Fluorescent-oligonucleotide probing of whole cells for determinative, phylogenetic, and environmental studies in microbiology. *J Bacteriol* 1990; **172**: 762–770.
49. Wallner G, Amann R, Beisker W. Optimizing fluorescent in situ hybridization with rRNA-targeted oligonucleotide probes for flow cytometric identification of microorganisms. *Cytometry* 1993; **14**: 136–143.
50. Thiele S, Fuchs BM, Amann RI. Identification of microorganisms using the ribosomal RNA approach and fluorescence in situ hybridization. *Treatise on Water Science*. 2011. Elsevier, pp 171–189.
51. Bennke CM, Reintjes G, Schattenhofer M, Ellrott A, Wulf J, Zeder M, et al. Modification of a high-throughput automatic microbial cell enumeration system for shipboard analyses. *Appl Environ Microbiol* 2016; **82**: 3289–3296.
52. Arnosti C. Fluorescent derivatization of polysaccharides and carbohydrate-containing biopolymers for measurement of enzyme activities in complex media. *J Chromatogr B Analyt Technol Biomed Life Sci* 2003; **793**: 181–191.
53. Fuchs BM, Pernthaler J, Amann R. Single cell identification by fluorescence in situ hybridization. *Methods for General and Molecular Microbiology*. 2007. ASM Press, Washington, DC, USA, pp 886–896.
54. Wang F-Q, Bartosik D, Sidhu C, Siebers R, Lu D-C, Trautwein-Schult A, et al. Particle-attached bacteria act as gatekeepers in the decomposition of complex phytoplankton polysaccharides. *Microbiome* 2024; **12**: 32.
55. Ondov BD, Treangen TJ, Melsted P, Mallonee AB, Bergman NH, Koren S, et al. Mash: fast genome and metagenome distance estimation using MinHash. *Genome Biol* 2016; **17**: 132.
56. Jain C, Rodriguez-R LM, Phillippy AM, Konstantinidis KT, Aluru S. High throughput ANI analysis of 90K prokaryotic genomes reveals clear species boundaries. *Nat Commun* 2018;

9: 1–8.

57. Li H. New strategies to improve minimap2 alignment accuracy. *Bioinformatics* 2021; **37**: 4572–4574.
58. Aroney STN, Newell RJP, Nissen JN, Camargo AP, Tyson GW, Woodcroft BJ. CoverM: read alignment statistics for metagenomics. *Bioinformatics* 2025; **41**: btaf147.
59. Nayfach S, Pollard KS. Average genome size estimation improves comparative metagenomics and sheds light on the functional ecology of the human microbiome. *Genome Biol* 2015; **16**: 51.
60. Grondin JM, Tamura K, Déjean G, Abbott DW, Brumer H. Polysaccharide utilization loci: Fueling microbial communities. *J Bacteriol* 2017; **199**.
61. Bjursell MK, Martens EC, Gordon JI. Functional genomic and metabolic studies of the adaptations of a prominent adult human gut symbiont, *Bacteroides thetaiotaomicron*, to the suckling period. *J Biol Chem* 2006; **281**: 36269–36279.
62. Shuoker B, Pichler MJ, Jin C, Sakanaka H, Wu H, Gascueña AM, et al. Sialidases and fucosidases of *Akkermansia muciniphila* are crucial for growth on mucin and nutrient sharing with mucus-associated gut bacteria. *Nat Commun* 2023; **14**: 1833.
63. Derrien M, Belzer C, de Vos WM. *Akkermansia muciniphila* and its role in regulating host functions. *Microb Pathog* 2017; **106**: 171–181.
64. Berkhout MD, Plugge CM, Belzer C. How microbial glycosyl hydrolase activity in the gut mucosa initiates microbial cross-feeding. *Glycobiology* 2022; **32**: 182–200.
65. Aramaki T, Blanc-Mathieu R, Endo H, Ohkubo K, Kanehisa M, Goto S, et al. KofamKOALA: KEGG Ortholog assignment based on profile HMM and adaptive score threshold. *Bioinformatics* 2020; **36**: 2251–2252.
66. Mistry J, Chuguransky S, Williams L, Qureshi M, Salazar GA, Sonnhammer ELL, et al. Pfam: The protein families database in 2021. *Nucleic Acids Res* 2021; **49**: D412–D419.
67. Yin Y, Mao X, Yang J, Chen X, Mao F, Xu Y. dbCAN: a web resource for automated

carbohydrate-active enzyme annotation. *Nucleic Acids Res* 2012; **40**: W445–W451.

68. Prjibelski A, Antipov D, Meleshko D, Lapidus A, Korobeynikov A. Using SPAdes DE Novo Assembler. *Curr Protoc Bioinformatics* 2020; **70**: e102.

69. Li D, Liu C-M, Luo R, Sadakane K, Lam T-W. MEGAHIT: an ultra-fast single-node solution for large and complex metagenomics assembly via succinct de Bruijn graph. *Bioinformatics* 2015; **31**: 1674–1676.

70. Alneberg J, Bjarnason BS, de Bruijn I, Schirmer M, Quick J, Ijaz UZ, et al. Binning metagenomic contigs by coverage and composition. *Nat Methods* 2014; **11**: 1144–1146.

71. Delmont TO, Eren EM. Linking pangenomes and metagenomes: The *Prochlorococcus* metapangenome. *PeerJ* 2018; **2018**.
